# Effect of Longevity Genetic Variants on the Molecular Aging Rate

**DOI:** 10.1093/geroni/igaa057.3130

**Published:** 2020-12-16

**Authors:** Paola Sebastiani, Anastasia Gurinovich, Zeyuan Song, William Zhang, Stefano Monti, Sofiya Millman, Nir Barzilai, Thomas Perls

**Affiliations:** 1 Boston University, Boston, Massachusetts, United States; 2 Albert Einstein College of Medicine, Bronx, New York, United States; 3 Boston University School of Medicine, Boston, Massachusetts, United States

## Abstract

We conducted a genome-wide association study of 1317 centenarians from the New England Centenarian Study and 2885 controls using >9M genetic variants. The most significantly associated variants were correlated to 4131 serum proteins in 224 study participants. The genetic and protein associations were replicated in a genome-wide association study of 480 centenarians and ~800 controls of Ashkenazy Jewish descent and a proteomic scan of approximately 1000 participants of the same study. The analysis replicated a protein signature associated with APOE genotypes and confirmed strong overexpression of BIRC2 (p < 5E-16) and underexpression of APOB in carriers of the APOE2 allele (p< 0.05). The analysis also discovered and replicated associations between longevity variants and slower changes of protein biomarkers of aging, including a novel protein signature of rs2184061 (CDKN2a/CDKN2B in chromosome 9). The analyses show that longevity variants correlate with proteome signatures that could be manipulated to discover healthy aging targets.

